# The challenges of changing national malaria drug policy to artemisinin-based combinations in Kenya

**DOI:** 10.1186/1475-2875-6-72

**Published:** 2007-05-29

**Authors:** Abdinasir A Amin, Dejan Zurovac, Beth B Kangwana, Joanne Greenfield, Dorothy N Otieno, Willis S Akhwale, Robert W Snow

**Affiliations:** 1 Malaria Public Health & Epidemiology Group, Centre for Geographic Medicine Research-Coast, Kenya Medical Research Institute/Wellcome Trust Research Programme, 00100 GPO, P.O. Box 43640, Nairobi, Kenya; 2 Centre for Tropical Medicine, University of Oxford, John Radcliffe Hospital, Headington, Oxford, OX3 9DU, UK; 3 WHO-Kenya, P.O. Box 45335-00100, Nairobi, Kenya; 4 Division of Malaria Control, Ministry of Health, 00100 GPO, P.O Box 20750, Nairobi, Kenya

## Abstract

**Backgound:**

Sulphadoxine/sulphalene-pyrimethamine (SP) was adopted in Kenya as first line therapeutic for uncomplicated malaria in 1998. By the second half of 2003, there was convincing evidence that SP was failing and had to be replaced. Despite several descriptive investigations of policy change and implementation when countries moved from chloroquine to SP, the different constraints of moving to artemisinin-based combination therapy (ACT) in Africa are less well documented.

**Methods:**

A narrative description of the process of anti-malarial drug policy change, financing and implementation in Kenya is assembled from discussions with stakeholders, reports, newspaper articles, minutes of meetings and email correspondence between actors in the policy change process. The narrative has been structured to capture the timing of events, the difficulties and hurdles faced and the resolutions reached to the final implementation of a new treatment policy.

**Results:**

Following a recognition that SP was failing there was a rapid technical appraisal of available data and replacement options resulting in a decision to adopt artemether-lumefantrine (AL) as the recommended first-line therapy in Kenya, announced in April 2004. Funding requirements were approved by the Global Fund to Fight AIDS, Tuberculosis and Malaria (GFATM) and over 60 million US$ were agreed in principle in July 2004 to procure AL and implement the policy change. AL arrived in Kenya in May 2006, distribution to health facilities began in July 2006 coincidental with cascade in-service training in the revised national guidelines. Both training and drug distribution were almost complete by the end of 2006. The article examines why it took over 32 months from announcing a drug policy change to completing early implementation. Reasons included: lack of clarity on sustainable financing of an expensive therapeutic for a common disease, a delay in release of funding, a lack of comparative efficacy data between AL and amodiaquine-based alternatives, a poor dialogue with pharmaceutical companies with a national interest in antimalarial drug supply versus the single sourcing of AL and complex drug ordering, tendering and procurement procedures.

**Conclusion:**

Decisions to abandon failing monotherapy in favour of ACT for the treatment of malaria can be achieved relatively quickly. Future policy changes in Africa should be carefully prepared for a myriad of financial, political and legislative issues that might limit the rapid translation of drug policy change into action.

## Background

In recent years the threat posed by failing, but inexpensive, antimalarial monotherapies led to an international effort to replace these drugs with relatively more expensive but considerably more effective artemisinin-based combination therapies (ACTs) for the management of uncomplicated malaria [1-3]. However, a change in international therapeutic recommendations does not always translate to an immediate, effective policy change at country levels.

Several authors have commented on the difficulties facing national antimalarial drug policy change when these changes involved moving from one inexpensive failing drug, such as chloroquine (CQ) to an equally widely available, inexpensive but more efficacious monotherapy such as sulphadoxine-pyrimethamine [4-8]. These observations during the 1990's and early 2000's highlighted the complexity of drug policy change and implementation for malaria case-management in Africa.

Since the inception of Roll-Back-Malaria (RBM) and the renaissance in malaria control and prevention in Kenya, the Ministry of Health (MoH) has had to change its first-line recommendations for the treatment of uncomplicated malaria twice. The first change occurred in 1998 when sulphadoxine/sulphalene-pyrimethamine (SP) replaced CQ. The processes leading to policy change and implementation have been described previously [4]. In 2004 the policy was changed again from SP to an ACT. This paper reviews the evidence used to effect this policy change and the political and economic challenges facing the Kenyan MoH prior to and during the implementation of the policy 32 months after the policy change was announced.

## Methods

All published and unpublished documentary evidence surrounding the antimalarial drug policy change over the period 2001 to 2006 were reviewed, including narrative reports from stakeholders involved in the drug policy change and implementation (obtained through the authors' informal networks), MoH reports, minutes of meetings, and email correspondence between actors in the policy change process. In addition, all articles related to malaria and the antimalarial drug policy were prospectively compiled from the two leading newspapers in Kenya (the Daily Nation and The Standard) for the same period. These data were supplemented by the authors' observations of the policy change process (several of the authors were actively involved and attended many of the relevant meetings). Data have been structured to capture the timing of events, the difficulties and hurdles faced and the resolutions reached to the final implementation of a new treatment policy. Several drafts of the final narrative were shared with stakeholders who clarified and corrected specific issues.

## Results

### Establishing the evidence and basis for drug policy change

Following the official transition from CQ to SP in 1998 the MoH's Division of Malaria Control (DOMC) and research partners maintained a series of surveillance studies on the sensitivity of SP and amodiaquine (AQ) [9,10], the recommended first and second-line treatments for uncomplicated malaria respectively. By 2001, the year the National Malaria Strategy was officially launched, concerns were raised about growing evidence of a decline in SP clinical efficacy as measured through the then standard WHO day 14 clinical and parasitological sensitivity test. By mid-June 2001, 6/15 (40%) studies undertaken by the DOMC and 3/6 (50%) by other partners showed that SP clinical and parasitological failure rates by day 14 were in excess of 25% (the WHO suggested change rubric of 25% failure rate is commonly used in the sub-region to inform antimalarial drug policy changes) [10-13]. Conversely, there appeared to be better day 14 cure rates (≥ 75%) across studies where AQ was tested (19/20 studies) [10,13]. In June 2001 a meeting was convened by the DOMC and its partners to discuss strategies principally around how better to deliver medicines through the retail sector. However, the meeting also raised the urgent need to assemble the evidence on SP failure rates noting that "...*plans for the introduction of a replacement [were] now urgent*..." [14]. A second meeting was held just four months later to "...*review the national antimalarial drug policy and build a national consensus on malaria treatment*..." [15]; however, it wasn't until the final quarter of 2003, that the status of SP was deemed desperate requiring the formation of a national task force [16-18]; at this time, seven out of nine studies (more than 75%) conducted between 2002 and 2003, including those examining patients through to day 28, showed SP failure rates in excess of 25% [10,19]. Whilst it was accepted that a change to a new first line therapy was urgently required, the possibilities for replacements were limited.

The decline in the clinical efficacy of SP in Kenya was happening within the context of an international push towards ACT in countries where monotherapies were failing [1,2,21] at a time when attention was focused on increasing international funding for effective malaria therapies [21]. The Global Fund to Fight AIDS, Tuberculosis and Malaria (GFATM) was launched in January 2002 as a financing mechanism for commodities and delivery of effective control of HIV/AIDS, TB and malaria. In January 2004, the publication of claims of "medical malpractice" by the GFATM for financing countries requesting drugs that weren't clinically efficacious heightened the debate on rapid deployment of ACT across Africa [2]. Kenya was included in this criticism as they requested in September 2002 approximately two million US dollars (US$) worth of SP in their successful Round-2 GFATM bid [22].

The first Kenyan Drug Policy Technical Working Group (DPTWG) meeting was convened on 6^th ^November 2003 [16]. The working group comprised the MoH, non-governmental organization (NGO) partners, bilateral donors, representatives of the research community and technical support from the World Health Organization (WHO). Data on SP and AQ sensitivity were reviewed and possible first-line replacements considered. Options considered were: moving AQ from second-line treatment to first-line therapy; combining SP with artesunate; using the recently registered chlorproguanil-dapsone (LAPDAP^®^) product; combining AQ with artesunate (AQ-AS); or using artemether-lumefantrine (AL). However, unlike the previous antimalarial drug policy change in 1998, there was a lack of any nationally generated, comparable sensitivity data, with the exception of AQ monotherapy, on the suggested alternatives. This posed a problem for informed choices by the DPTWG. The DPTWG felt that given the current pressure to avoid monotherapy and the short-term gains in combining SP with another partner drug eliminated these choices. Further, the WHO was unhappy about wide scale use of LAPDAP^® ^without further safety data [23]. Thus, the choices were between AQ-AS versus AL. The need to assemble more information on AQ sensitivity and undertake carefully conducted open label, day 28 sensitivity studies of AQ-AS and AL were recommended to fill the information void.

There was only one clinical efficacy study undertaken in Kenya on AL available for review pre-publication in January 2004. This study was undertaken at Kilifi, on the Kenyan coast, as part of multi-country Phase III regulatory approval studies for the 6-dose AL recommendation [24]. The interim results showed that over 95% of children attained adequate clinical and parasitological response by day 28 for both the supervised and unsupervised groups. The data generated from this study were presented to the DOMC during the 3^rd ^DPTWG who felt that one study was insufficient and insisted on a multi-site comparison of AL with AQ-AS. Deliberations on who would undertake these studies and how they would be funded continued through the 4^th ^and 5^th ^DPTWG meetings between January and March 2004 [13,19]. These studies were never completed. WHO provided technical support to the DOMC and provided examples of other countries, such as Zanzibar and Zambia, who changed policy using only international and regional evidence without comparative national data [23]. The WHO also encouraged the DPTWG to consider the operational complexities of carrying out drug policy change and that issues of implementation should be defined early. In January 2004 the DOMC formed sub-groups of the DPTWG to address specific drug and non-drug issues related to the drug policy change, including therapeutic efficacy testing; legal issues; guidelines and formulations; logistics, procurement and supplies; case management; and later Information, Education and Communication (IEC) [13].

Debate continued over the quality and amount of evidence available on the efficacy of AQ. Most of the day 28 studies undertaken by the DOMC under the auspices of the East African Network for Monitoring Antimalarial Treatment (EANMAT) were not corrected for possible new infections and that these data were at variance with data generated by the Africa Medical Research Foundation (AMREF) at other sites that showed high failure rates for AQ monotherapy [13]. These discrepancies were never fully resolved and a view persisted that AQ was a valuable long-term combination partner by some members of the DPTWG despite evidence of AQ failure from neighboring countries [10].

On the 3^rd ^March 2004 an urgent meeting of the DPTWG was convened in anticipation of the deadlines for the Round 4 GFATM application on the 5^th ^of April 2004 [19]. It was clear that a decision had to be made on what ACT was to be announced as the replacement therapy for SP, an estimation of drug needs and costs was required and a strategy for delivery articulated and costed. A decision based on international, regional, sub-regional and country efficacy data and country experiences was taken at this DPTWG meeting in favour of AL, with oral quinine recommended as the second-line treatment. The decision was based largely on issues related to AL being the only co-formulated ACT at the time, doubts that AQ could be withdrawn from the informal sector as a monotherapy while deploying AQ-AS in the formal sector and the assumed rising levels of existing AQ resistance across Kenya. Concerns were, however, raised about the high cost of AL despite subsidized arrangements between WHO and Novartis Pharma AG [3], and the notion that the global supply of artemisinin was in jeopardy [19]. Both issues affected both AQ-AS and AL. It is notable that at the time of the final decision to adopt AL there were no comparative efficacy data on AL versus AQ-AS nor any substantiated DOMC generated data on day 28 AQ sensitivity.

On the 5^th ^April 2004 the "National Symposium on Next Anti-Malaria Treatment Policy in Kenya" was held at Naivasha. The DPTWG sub-committees and other key participants summarized their deliberations before the Minister for Health informed the gathering that, after negotiations with relevant bodies and development partners, Kenya had opted to change policy to the WHO recommended ACT and AL was now the recommended first-line treatment for uncomplicated malaria [25]. The new policy was reiterated on the 25^th ^April 2004, Africa Malaria Day, in a speech delivered by the Deputy Director of Medical Services [26]. From this point began the long process of policy implementation.

The GFATM Round-4 malaria proposal was submitted on the 5^th ^April 2004 and approved by the GFATM Technical Review Panel (TRP) in July 2004 amounting to over 188 million US$ over 5 years including key components of implementation of the new drug policy and scaling up insecticide treated net coverage. The funds were approved to be disbursed in two phases, the first amounting to over 82 million US$, including approximately 40 million US$ to cover the costs of procuring approximately 11 million treatment doses of AL annually; second, the balance of funds to be accessed following successful implementation of the first phase [27,28]. Kenya proposed to phase in the new drug policy, beginning with a two year (2006–2007) introduction of free distribution of AL through the public formal sector (government, NGO and mission health facilities), followed by its use within the private formal sector from 2008 and finally scaling-up to facilitate distribution through the private for-profit retail sector from 2009. The initial three year restriction to the formal sector was proposed as a way to build national confidence in the new drug and to establish a better pharmacovigilance profile before deregulating use for over-the-counter (OTC) in 2009. During this period, it was proposed that AQ be promoted as an alternative to AL in the retail sector [29].

In summary, the decision to replace SP with AL as the nationally recommended therapy for uncomplicated malaria was a pragmatic choice. It was based on limited comparative scientific data (between AL and competing alternatives such as AQ-AS); legitimate international pressure to abandon as soon as possible drugs that were no longer efficacious; and the opportunity to fund more expensive medicines through an international financing mechanism. The GFATM announced on the 1^st ^July 2004 that Kenya had been successful in its application for US$ 82 million for phase 1 of Round-4. However, the practicalities of a rapid translation of policy into practice were only fully appreciated after the policy was announced.

### Delays in translating policy into practice

Despite several consultative meetings of the DPTWG and other WHO advisory meetings, an announcement by the Minister for Health and a successful application to the GFATM, there remained several concerns about the implementation of the recommendation to adopt AL as first line therapy. These revolved around long-term predictable financing, procurement and supply, ensuring adequate access across all service providers, the concerns of pharmaceutical manufacturers, and regulatory issues with regard to widening access to AL.

#### Financing, procurement and supply challenges

Concerns were raised regarding the long-term financial sustainability of the new policy, especially in light of the recent MoH's experience with the *Haemophilus influenzae *type B (Hib) vaccine. Hib was introduced in the country in 2001 [30] with an initial funding commitment from the Global Alliance for Vaccines and Immunization. It was initially agreed that at the end of the five-year funding cycle in 2006, the MoH would incorporate Hib in the Expanded Programme for Immunization. The agreement was based on optimistic estimates that the cost of the vaccine would come down substantially [30,31]. However, this did not happen and the MoH found itself saddled with an expensive intervention for which it did not have money. In light of this, the MoH viewed AL as an expensive first-line antimalarial drug replacement, costing at least ten times more than SP at the proposed subsidized prices, which could not be financed directly and solely by the MoH. In the absence of external funding, the cost of AL alone would absorb the entire MoH budget for rural drug supply [32-35].

In June 2004, the Director of Medical Services (DMS) requested an economic evaluation of *not changing policy*. In September 2004, the DMS called for an urgent meeting to discuss the AL drug policy decision where it was resolved that an official assurance should be sought from the GFATM that the new policy would not be jeopardized over the next five years because of a failure after two years to meet all the milestones set for implementation of non-drug policy related funding requests [32]. A few weeks later, on 30^th ^September 2004, a meeting was called by the GFATM to offer countries technical assistance on re-programming committed funds from Round-2 funding for monotherapies. The Kenyan DMS asked for an unequivocal assurance on continued funding, however the GFATM maintained that it was unable to provide this assurance given the vagaries of its own donors [36].

The economic analysis became an ever increasing concern for the MoH and was discussed at length during subsequent meetings of the DPTWG during the delay in signing the GFATM Round 4 agreement [37,38]. Some bilateral donors such as the UK Department for International Development (DFID) tried to allay the fears of financial sustainability by pledging funds to kick-start the purchase of AL on one hand and trying to lobby long term financial support for the policy from other partners on the other. The economic analysis was never undertaken and decisions to continue with AL were taken without this analysis.

Another challenge facing the DOMC and MoH was navigating the complex financial and procurement arrangements for AL [39-42]. The main challenge centered on how to manage the financial flows to make sure funds were availed in time for orders to be placed and processed. Theoretically, funds would flow from the GFATM, to the principal recipient (Ministry of Finance), then to the MoH (sub-recipient). The MoH would, after consultation with the national procurement consortium established to manage the tendering and ordering of commodities purchased with GFATM funds, place an order with WHO to forward the order to the supplier (Novartis Pharma AG in Switzerland). As per GFATM rules a Local Fund Agent (LFA), KPMG Kenya, should verify disbursement requests and financial reports on behalf of the GFATM. Quarterly release of monies already approved under Rounds-2 and 4 for malaria were contingent on the LFA signing off on how well the funds disbursed during the previous quarter were used; this, in turn, was used as a proxy for meeting performance targets. Although the GFATM TRP approved the Round-4 proposal in July 2004, concerns were raised by the GFATM about meeting broad performance targets, local fund management and procurement related to funds allocated for HIV/AIDs, TB and malaria during Round-2. This delayed considerably the final signing of the Round 4 agreement which happened 9 months after the TRP approval [43].

Reconciling the complexity of the ordering and procurement procedures delayed the final submission of the AL request to WHO. The request was finally made in June 2005 and approved by WHO within two days. In addition, the delays in flow of Global Funds continued to postpone the timing of programmatic activities such as health worker training on the new drug, planned around the drug's arrival in the country. To circumvent the complex process of paying for the drugs once the order had been placed, a decision was made by the MoH to have the GFATM directly pay Novartis Pharma AG for AL orders but required the approval of the Ministry of Finance (MoF). The MoH wrote to the MoF in September 2005 and approval for this arrangement was obtained in December 2005, six months after an order had been initially placed with the WHO. Following the signed GFATM Round-4 agreement and a means to disburse the funds to WHO, the original order was processed in February 2006 and the first AL supplies arrived in Kenya three months later. The process was reduced in subsequent orders to only three months from placing an order receipt, financial disbursement, to arrival of supplies in country.

#### National and international pharmaceutical interests

On 8^th ^April 2004, three days after the AL policy change was first announced, a regional meeting on AQ-AS was launched for approximately 13 francophone countries (which were using the product) with Kenya as the host. The publicity surrounding this meeting overshadowed Kenya's own policy change announcement. This caused confusion, especially in the media, as to what the new first-line policy was. Between April 8^th ^and 13^th ^2004, media reports appeared to endorse AQ-AS as the first line drug for malaria [44-46]. These reports were denied by the then head of the DOMC [47], but confusion continued in the media where reports in the print media supported the use of competing artemisinin products, such as artesunate-mefloquine [48] and even a two-day regimen of dihydroartemisinin-piperaquine [49].

The special arrangements between WHO, Novartis Pharma AG and GFATM posed a problem under the notion of single sourcing of drugs, contrary to the public sector procurement rules that insisted upon competitive bidding for government contracts unless a case could be made for an exception [50]. Even though WHO was the broker for AL supply, the tender process, paradoxically still had to be adhered to. This was highlighted in a claim by the Pharmaceutical Society of Kenya (PSK) in the national press accusing the WHO of a "monopoly" [51,52]. Tender documents, therefore, had to be prepared by the national GFATM procurement consortium in January 2005, with the final drafts sent to the Permanent Secretary, MoH, by February 14^th ^2005 and placed in the local newspapers (February 18^th ^2005) for the supply of malaria drugs [53-55]. This insistence on adhering to the letter of the law even when there was a clear national need for an exception, further delayed the procurement of AL by the MoH.

The PSK made several representations through letters and position papers to the MoH, presentations to media houses, and organizing workshops questioning the choice of AL as first-line between August 2004 and April 2005 [35,56]. The DOMC explained its position in a rejoinder to the PSK in September 2004 [32,57] stating the technical reasoning behind the selection of AL. This did not change the position of PSK and its members, who have maintained their strident opposition to the new policy to date. There are large stocks of artemisinin monotherapies, SP and AQ in the Kenyan market [58] and concerns among local manufacturers and importers of pharmaceuticals of losing a market share to a transnational company.

#### Regulatory issues regarding widening access to AL

AL remains a prescription-only-medicine (POM) in line with all artemisinin monotherapies and other combinations [34]. For it to be availed in the non-premium, private retail sector, where a substantial number of Kenyans seek treatment for malaria fevers [59], requires mechanisms to deregulate its POM status. The policy implementation plan acknowledges the need to deregulate AL for OTC use and develop some evidence based operational research to maximize the best use of this drug as an OTC medicine [60]. However, theoretically the POM status would only be changed following the assembly of adequate Phase IV safety data. The Pharmacy and Poisons Board (PPB) will launch a pharmacovigilance system that will entail passive reporting of adverse reactions to all medicines, including AL and other antimalarial drugs, in the second quarter of 2007 (Mohamed A, personal communication).

The POM status of AL also affected its effective deployment in the formal health sector. According to the Pharmacy and Poisons Act, not all cadres of health workers can prescribe or dispense POM drugs; only medical doctors, dentists, clinical officers and graduate pharmacists (in emergencies only) are allowed to prescribe. Graduate pharmacists can dispense all prescription drugs, but pharmaceutical technologists can only dispense Part II poisons (the so-called pharmacy only drugs); Part I poisons can only be dispensed under supervision of a graduate pharmacist [61]. This obviously posed a problem for lower cadre health workers, such as nurses, who manage the majority of malaria prescriptions and dispensing in most government and mission health facilities. The Act invests many powers in the Minister of Health who can make exceptions to the rules and subsidiary legislation for better service delivery. Normally, the MoH would issue a legal notice through the Kenya Gazette to cover such exceptions, but this has not happened to date.

### National implementation of the revised drug policy

Recognizing the complexities of implementing a new drug policy, based on experiences of moving from CQ to SP, the DOMC configured several sub-committees from the DPTWG as early as March 2004. Their mandates were to revise existing guidelines and make recommendations on the best strategies for implementing the new policy [19]. The process by which these recommendations from these committees were made and their effects on implementation of the AL policy are discussed below.

#### Revising national treatment guidelines

The first task was to update the "*National guidelines for diagnosis, treatment and prevention of malaria for health workers in Kenya*", last revised in 1998 [62]. A series of draft guidelines were developed following an initial draft in August 2004. The guidelines were finalized in March 2006 [63]. This process took approximately 23 months to complete following the official announcement of the new AL policy in Kenya. Delays were frequently encountered at each iteration of the revised guideline beginning with uncertainties surrounding the policy recommendation (described above) and was characterized with ambiguities on how to word the policy with some arguing for a general term such as "ACT" and not "AL". Consensus was finally achieved and AL was explicitly stated as the first line treatment for uncomplicated malaria. Additional delays were encountered in reaching a consensus on how to eliminate ambiguous messages on the interpretation of negative diagnostic tests from the previous guidelines, the incorporation of information and interpretation of new rapid diagnostic tests, the need to harmonize recommendations for children below five years of age with fever algorithms of the Integrated Management of Childhood Illness guidelines [64] and the development of new algorithms for outpatient case-management for febrile patients above five years of age. All these revisions required multiple consultations with many stakeholders and different divisions within the MoH. Finally, between March and July 2006, 8,500 copies of the revised guidelines were produced and subsequently disseminated to health workers through the district health management teams and during in-service trainings.

#### In-service training on revised national treatment guidelines

The second task was to train health workers on the new case management guidelines. Following completion of the guidelines in March 2006, the DPTWG developed facilitators and participants manuals for in-service training [65,66]. The training was organized in a cascade manner with the first training of trainers (TOT) workshop held for 32 participants in April 2006 in Nairobi [67]. A second national TOT was held in May 2006, combined with a national policy dissemination workshop. A total of 48 participants were trained, including provincial medical officers, representatives from the Christian Health Association of Kenya, the Kenya Episcopal Conference (representing missions facilities run by the Catholic Church), staff of the Mission for Essential Drugs and Supplies (MEDS), representatives from major private hospitals in the country and those from professional bodies such as PSK and the Kenya Medical Association [68]. Provincial level training was undertaken in June 2006 with approximately 405 TOTs trained, mostly members of District Health Management Teams and senior district hospital staffs [69]. A series of district level trainings for health workers were launched between August and October 2006 and included mostly prescribers at lower ends of the health system: nurses and clinical officers, laboratory technicians, public health officers and pharmacists. At all levels of the training cascade, the training was organized in the form of 3-day workshops following the same curriculum for approximately 30–40 participants per training session. The cascade in-service training cost approximately 1.47 million US$ and was planned to cover over 60% of front-line health workers countrywide within three months to ensure training was completed before the delivery of AL to rural health facilities. In practice, however, there were delays in the release of GFATM funds to support the district-level training which inevitably resulted in some facilities in some parts of the country receiving AL without in-service training in its use. However, support was received from WHO and DFID-UK to bridge funding gaps for training and full roll out of the district-level training activities was completed in all districts by December 2006 covering approximately 9,000 government and mission employed health workers including those responsible for treatment, pharmacy and laboratory services. In addition, approximately 236 private practitioners and health workers in some government parastatals and the armed forces were made aware of the new drug policy during workshops organized by the Management Sciences for Health between August and October 2006.

#### Logistics, procurement and supplies

An implementation plan for the new policy was established by July 2005 with logistics and supply chain management as one of the crucial components [29]. The initial June 2005 order followed a quantification exercise that estimated that on average 10 million treatment courses of AL would be required annually [28,29,70]. Orders were initially to be split into two 5 million treatment courses to be received six months apart, however, because of the short shelf-life of AL (24 months), the delivery of AL was staggered quarterly with the first delivery of 2.63 million treatment courses received in-country on the 18^th ^May 2006. The delivery for the next quarter was split into two with the first shipment of 1.30 million treatment courses arriving in the country in August 2006, and the second shipment of 1.11 million treatment courses arrived in November 2006. Because of a drop in the international prices of AL announced by Novartis Pharma AG in September 2006 [71], the drug supply for the second half of 2006/07 changed from the anticipated 5 million to 7.5 million treatment courses. The first part of the consignment (3.2 million treatment courses) arrived in January 2007 and the balance arrived in Kenya in March 2007 [72].

Drugs were received by the Kenya Medical Supplies Agency (KEMSA), who had the responsibility to distribute AL directly to facility levels and guarantee drug availability immediately post-in-service training. KEMSA was allocated approximately 70% of the AL supply for distribution to government facilities and 30% were allocated to MEDS for distribution to approximately 850 mission facilities. Drug distribution from KEMSA began in July 2006. Under the distribution schedule all facilities in North Eastern and Coast province were expected to request supplies based upon consumption each month (a "pull system"). All hospitals (provincial, district, sub-district) and some health centres nationwide were similarly expected to make monthly supply requests. Non-hospital facilities in all other provinces were expected to be supplied using a "push system" of standard drug kits every three months. To support on-going quantification of drug needs supplies are accompanied with a new stock and inventory management system that should feed back to the DOMC and KEMSA to re-adjust future orders and supplies of AL.

#### Community awareness campaigns

A key strategy for the success of the new policy was a clear and well articulated IEC programme. In April 2006 the "Advocacy and Public Awareness Campaign For Artemisinin Combination Therapy (ACT) In Kenya" plan was developed [73]. This plan recognized that the comparative advantage for mobilizing wide-scale public support for the new drug policy was with the private-for-profit marketing sector. A tender was issued for IEC services to implement the strategy, and awarded to a local advertising company in June 2006 [74]. The approach was multi media including print media advertisements (10 spots), television (481 spots), national and regional vernacular radio (3,064 spots) and community road shows predominantly in Kiswahili. Approximately 100,000 posters and 500,000 brochures were distributed countrywide to educate the public on the burden of malaria and to reinforce key messages on the appropriate first-line drug including where and how to access AL free-of-charge [74]. The IEC campaign was provided important legitimacy when it was officially launched by the President in September 2006 under the slogan *Komesha Malaria, Okoa Maisha *(Stop Malaria, Save a Life) [75]. The IEC campaign ran for over three months, costing approximately 0.48 million US$ and planned to reach 60% of the primary target audience, caregivers of children under five years of age.

**Figure 1:**
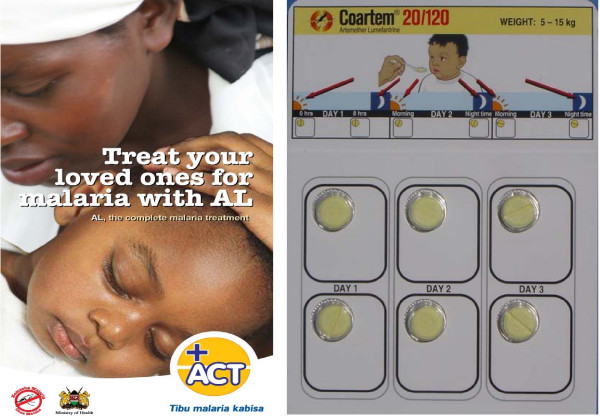
Figure showing public promotion of the generic terms "ACT" and "AL" (left panel) and labeled drug provided to patients (right panel).

A key IEC challenge is product branding. Currently the print and electronic media carry the message that the first-line antimalarial drug policy in Kenya is ACT, and AL is the ACT recommended in Kenya, to be provided free of charge at government and mission health facilities [75]. However, the product procured through the Novartis Pharma AG-WHO arrangement is branded Coartem^® ^and that is what patients receive at public health facilities (Figure 1). The MoH have taken a legitimate position that they cannot be seen to be promoting a single product brand; however this difference in labeled IEC messages and products dispensed might lead to some confusion by the patient population.

## Discussion

It is acknowledged that in assembling the evidence a few milestone dates and discussions between key actors may have been missed. However, on balance, the narrative identifies the complexity of the drug policy change decision and implementation. The description of Kenya's policy change raises a number of issues for the future implementation of any new ACT drug policy. First, the strident opposition from the local pharmaceutical industry through such professional bodies as the PSK was not entirely without merit. One of the key strategies of ensuring sustainable drug supply at national levels, and defined in the Kenya National Drug Policy [76], is local manufacture of pharmaceuticals. There was a feeling within local pharma that they were not adequately engaged in the policy change process and that real debate actually begun after the policy was announced in Naivasha by the Minister of Health. Legitimate questions such as what to do with their huge stocks of SP and AQ and currently the artemisinin monotherapies have not been adequately addressed.

**Figure 2:**
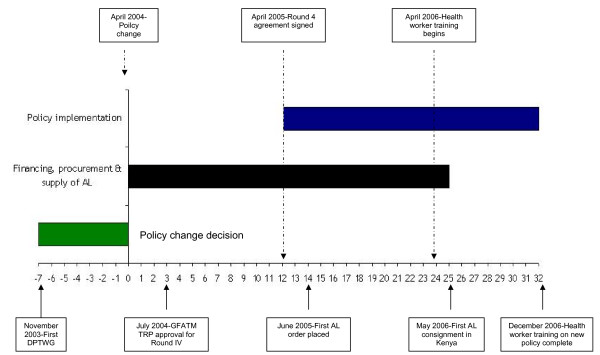
Timeline of key events in the policy change implementation in Kenya: The green bar represents the time taken from the first drug policy technical working group meeting to consider policy options to the announcement by the Ministry of Health of the decision to adopt AL as the first-line therapeutic. The black bar represents the protracted time taken following the drug policy announcement to secure international financial support, order the drug and the drugs arrival in Kenya. Finally the blue bar represents the time taken from the release of funding to completion of the implementation of the drug policy change by December 2006.

Second, unlike the long delay (seven years) in taking the decision to abandon CQ following overwhelming local research evidence [4], the decision to abandon SP was swift, largely driven by a very vocal international malaria research community (Figure 2). This community had consistently argued for the rationale of ACT for malaria in Africa [3,77-80] and identified cost as the major impediment to having effective medicines deployed on the continent [21]. They, therefore, lobbied for more and better targeted international funds for priority diseases such as HIV/AIDS, TB and malaria [2,81]. However, choosing a replacement to SP in Kenya was based on less national evidence than when a decision was made to replace CQ with SP. This inevitably weakened the stakeholder's acceptance of the choice made and levels and future impacts of AQ resistance should have been better documented. It has subsequently emerged that levels of AQ resistance by day 21 are as high as 20% in some parts of Kenya [82] and similar credible data would have helped in defending the pragmatic decision made to transition to AL in April 2004.

Third, following a protracted decision to adopt SP as first line therapy in August 1998, the financing and implementation of the policy decision was much faster compared to the decision in 2004 to adopt AL. It took over two and half years from a technical decision being reached to support the use of AL as a first-line therapeutic to its actual arrival in country and deployment to health facilities with trained health workers supported by revised national treatment guidelines (Figure 2). The factors that resulted in a paralysis from policy decision to policy implementation included (1) concerns and government procurement difficulties with a single-sourced product; (2) timely access to external funds provided by the GFATM; (3) lack of agreement on whether there was a long-term, sustainable financing plan; and (4) competing local and international interests for alternatives to AL. The GFATM was in its nascent stages and experience needed to be built in putting up structures that would ensure its monies were spent in a transparent way. Nonetheless, alternative mechanisms of financing drug procurement require further attention and the issue of long-term financing remains unresolved.

Fourth, the legislative issues surrounding the use of new medicines at different levels of the formal and informal health sector are necessarily strict. However, flexibility is required to ensure that those who see patients most regularly within a formal health care setting can actually prescribe common medicines. For AL to be used an an OTC medicine in Kenya a functioning and effective pharmacoviligance system is required to guide legislation around this and other new ACT drug products. To a large extent these are non-existent in most countries in Africa [83]. Unless quality information on adverse event profiles can be built up around AL, this will present the Kenyan MoH with a difficult decision to make AL available OTC in 2009.

Finally, countries who have had to change their national antimalarial drug policy will recognize the complexities of harmonizing various national treatment guidelines, developing effective in-service training, ensuring adequate drug supply and educating the patient population. These activities consume huge amounts of ministry staff time and demand inputs from many other partners. The national treatment guidelines took over 23 months to revise, the cascade training took nine months to complete and while drug distribution from central stores to facilities was relatively quick it remains to be seen how a revised facility, pull-based ordering system will operate in the future. These activities must be carefully managed to avoid health workers being confronted with new drugs without information on how to prescribe and dispense them, trained health workers without the resources to implement the new policy or a patient population told that old medicines don't work but who cannot access new medicines. Despite a long delay in the implementation of the new policy, at an immeasurable cost to patients treated with failing drugs for two years, when orders were finally financed the MoH was prepared for implementation. The effectiveness of implementation is currently being evaluated as part of detailed studies of health worker and health facility performance. This step is critical because policy change and implementation does not necessarily translate into adequate quality case-management of patents at the point of care [84].

The early implementation of the revised malaria case-management strategy has many hurdles ahead. How to sustain regular drug supplies, manage new systems of drug ordering from health facilities to manufacturer, capitalize on the availability of effective medicines to improve the way patients are managed in the formal health sector, build a credible evidence-based platform of pharmacovigilance and use these data to inform the final endpoints of the policy to deregulate the POM status of AL for OTC use are all challenges yet to be tackled. These final stages of the policy change will require careful management by all stakeholders supported by credible operational research.

## Competing interests

The author(s) declare that they have no competing interests.

## Authorship statement

AAA, DZ, BBK, and RWS collected, collated, and interpreted the documents used in this paper and took part in drafting the manuscript; DNO and WSA were instrumental in documenting the minutes and official documents at the Ministry of Health. They also took part in drafting the manuscript, especially with regard to the policy implications of our key findings. JG provided information on elements of the policy implementation process and substantially revised the manuscript.
